# A Double-Layer Blockchain Based Trust Management Model for Secure Internet of Vehicles

**DOI:** 10.3390/s23104699

**Published:** 2023-05-12

**Authors:** Wenbo Ruan, Jia Liu, Yuanfang Chen, Sardar M. N. Islam, Muhammad Alam

**Affiliations:** 1 School of Cyberspace Security, Hangzhou Dianzi University, Hangzhou 310018, China; 212270057@hdu.edu.cn (W.R.); liujia@hdu.edu.cn (J.L.); 2 Key Laboratory of Discrete Industrial Internet of Things of Zhejiang Province, Hangzhou 310018, China; 3 Institute for Sustainable Industries and Liveable Cities (ISILC), Victoria University, Melbourne, VIC 3030, Australia; sardar.islam@vu.edu.au; 4 School of Engineering, London South Bank University, London SE1 0AA, UK; alamm52@lsbu.ac.uk

**Keywords:** Internet of Vehicles, blockchain, trust management, logistic regression

## Abstract

The Internet of Vehicles (IoV) enables vehicles to share data that help vehicles perceive the surrounding environment. However, vehicles can spread false information to other IoV nodes; this incorrect information misleads vehicles and causes confusion in traffic, therefore, a vehicular trust model is needed to check the trustworthiness of the message. To eliminate the spread of false information and detect malicious nodes, we propose a double-layer blockchain trust management (DLBTM) mechanism to objectively and accurately evaluate the trustworthiness of vehicle messages. The double-layer blockchain consists of the vehicle blockchain and the RSU blockchain. We also quantify the evaluation behavior of vehicles to show the trust value of the vehicle’s historical behavior. Our DLBTM uses logistic regression to accurately compute the trust value of vehicles, and then predict the probability of vehicles providing satisfactory service to other nodes in the next stage. The simulation results show that our DLBTM can effectively identify malicious nodes, and over time, the system can recognize at least 90% of malicious nodes.

## 1. Introduction

With the popularity of 5G and 6G [1] and the application of programmable V2X environments and blockchain-based V2X (vehicle to everything) technologies [2], the IoV has embraced rapid development. In IoV, vehicles can share their perceived information with other nodes, including traffic safety information, weather information, road information, etc., and obtain services from other nodes [3], thus, improving traffic safety and efficiency.

However, vehicles can be unreliable, and we need to solve the problems of how to evaluate the reliability of the message sent by the vehicle and quantify an evaluation measure [4] (i.e., trust value) based on the historical behavior of the vehicle before utilizing IoV. For example, in IoV, vehicles may be controlled by attackers to spread false information for selfish reasons, thus, leading to false environmental perception and driving decision-making and thus, endangering the safety of drivers and causing serious traffic accidents [5].

In IoV, the basic principle behind the trust model is to ensure the reliable transmission of data by identifying and canceling malicious vehicles and the false news generated by them [6]. The trust management mechanism can help vehicles calculate the credibility of received messages [7] to improve the accuracy of vehicles in decision-making. In summary, the existing trust management mechanisms can be generally divided into centralized trust management and distributed trust management [8]. Centralized trust management has problems such as single points of failure, while distributed trust management has problems such as the delayed update of trust value.

Blockchain, as bitcoin’s core technology, is a distributed ledger [9]. Due to its decentralized and immutable characteristics, blockchain can record and update vehicles’ trust values. With blockchain, even if a small number of RSUs have storage errors or are controlled by attackers, the consensus results of the entire network can still be protected. Therefore, some researchers combine blockchain with trust management mechanisms to solve the above problems of centralized and distributed trust management.

However, there are still some problems in the research of trust management mechanisms based on the blockchain or single-layer blockchain. First of all, the vehicles need to store a complete blockchain ledger or send a request to the adjacent full node for verification every time the transaction is verified, which will undoubtedly increase the burden of the vehicle and waste the vehicle’s resources. Secondly, because the number of blockchain nodes is very large and the coverage is active and wide, it is also difficult to conduct hierarchical management according to objective factors such as geographical location, communication traffic, and node density. Finally, because the importance of vehicle data is not the same, the data storage and data sharing between vehicles and RSUs is inefficient if the system does not distinguish the importance of messages. Therefore, how to enable the system to store and share data of different levels of importance is a problem. To solve the above problems, we propose a trust management mechanism based on the double-layer blockchain.

The main contributions of this paper are as follows:

1. We propose a double-layer blockchain trust management system for the Internet of Vehicles, including vehicle and RSU blockchain. Our DLBTM can selectively store messages according to their importance and provide accurate message services for vehicles;
2. We propose a trust evaluation method for vehicle nodes, including the message trust value of vehicles, the evaluation trust value of vehicles, and the historical trust value of vehicles. Then use logistic regression to update the above three trust values;
3. We use simulation experiments to prove the safety and effectiveness of DLBTM, and show that DLBTM can effectively distinguish malicious nodes from normal nodes.

## 2. Related Work: A Critical Literature Review

In this section, we mainly discuss the related work of centralized trust management, distributed trust management, a combination of blockchain and trust management, and a combination of double-layer blockchain and trust management.

### 2.1. Centralized Trust Management

Mahmoud et al. [10] adopted an incentive and punishment strategy (TRIPO) to prevent intentional packet loss attacks in rational cases and unintentional packet loss attacks in irrational cases. TRIPO uses small payments to reward rational nodes that correctly forward packets from other nodes. For irrational nodes, TRIPO uses a reputation system to measure, i.e., a new monitoring technique to monitor the nodes. However, all of these operations are centralized in the offline trusted party. Based on the malicious behavior detection system running on vehicles and RSUs, Bißmeyer et al. [11] proposed a centralized trust management model, which uses the malicious behavior report to establish trust relationships and reach the goal of identifying and removing attackers in IoV. Li et al. [12] proposed a reputable ad hoc network announcement scheme that consists of a centralized reputation server, access point (physical wireless communication equipment), and vehicle. The centralized reputation server’s role is to collect and aggregate feedback to generate reputation and spread reputation. The access point acts as the communication interface between the vehicle and the reputation server, and the vehicle broadcasts and receives information from neighboring vehicles. The credibility of the received information is evaluated and then reported to the reputation server.

All of the above schemes are managed by a trusted centralized server, which is vulnerable to attacks and expensive to maintain. In addition, centralized servers suffer from poor horizontal scalability, a single point of failure, and increased latency when nodes generate too many requests.

### 2.2. Distributed Trust Management

To solve the above problems, some researchers put forward distributed trust management models. Huang et al. [13] proposed a distributed reputation management system (DREAMS), in which basic reputation management tasks are performed by local authorities (LA) in different locations. LA acts as the trusted authority and arranges the vehicle edge computing server for local reputation display and updates. Oluoch et al. [14] also proposed a reputation model to help vehicles in the network evaluate the reliability of other vehicles, that is, each receiving vehicle requests other vehicles within its communication range to give reliability to the sending vehicle, or the receiving vehicle obtains the corresponding results from the RSU. Raya et al. [15] proposed a data-centric trust management model, which calculates the trust of each data, aggregates multiple related but possibly contradictory data, and finally obtains the final trust value.

The above studies all use distributed trust management models, which meet the requirements of distributed scenarios. However, these studies are not entirely distributed because most of the above studies have one or more central servers to store or process trust values, which goes against the main feature of distribution. Some studies also use vehicles or RSUs to store or process trust values. However, due to the limitations of the hardware conditions of vehicles and RSUs, security cannot be fully guaranteed. Moreover, due to the high-speed mobility of vehicles, trust values may not be updated in time, which may lead to serious consequences. For instance, the trust value decreases when a vehicle conducts malicious behavior in one area. However, due to the delay in updating the trust value when it drives into another area, the system treats the vehicle as a normal node, which may cause serious consequences to other nodes of the system. Therefore, how to build an efficient, secure, and fully trusted trust management model is an urgent problem to be solved.

### 2.3. Combination of Blockchain and Trust Management

The blockchain is decentralized, secure, anonymous, traceable, tamper-proof, etc., and has a wide range of applications in the IoV and other fields. Yang et al. [16] proposed a decentralized trust management model for IoV based on blockchain technology. The receiving vehicle uses Bayesian inference to verify the results for the messages received from adjacent vehicles. Then according to this result, the receiving vehicle generates scores for each vehicle sending messages and uploads them to the nearby RSU, which is responsible for calculating the variation of trust value of each vehicle according to the scores and packaging these data into a “block”. RSUs compete to become miners using the POW Consensus algorithm. Zhang et al. [17] proposed a trust management system for the IoV based on blockchain, which solves the problem of calculating message credibility. Moreover, this system can detect vehicles sending malicious messages and reduce their credit value according to the rating mechanism. In addition, a combination of the consensus mechanisms of PoW and PoS is used to ensure that vehicles with significant changes in reputation can be updated to the blockchain more quickly. Kang et al. [18] proposed a credit-based data sharing scheme, which considers the three weights of interaction frequency, event timeliness, and trajectory similarity, adopting the three-weight subjective logic (TWSL) model to select more reliable data sources and improve data credibility. In addition, the alliance blockchain is utilized to establish a secure and distributed vehicle blockchain and smart contracts are deployed on the vehicle blockchain to realize safe and efficient data storage of RSUs and data sharing among vehicles.

The above schemes solve the security of trust management, node trustworthiness, and other problems to a certain extent, but the consensus algorithm in [16,17] is inefficient and wastes a lot of computing power because it does not take comprehensive consideration when calculating message credibility. For instance, [16] only considers the distance between two vehicles to calculate received message credibility and [17] only considers the trust value of vehicles and the distance between two vehicles in the process of calculating message credibility. Therefore, the trust value calculated by this message credibility cannot reflect the historical behavior of vehicles well and, therefore, cannot further predict the future behavior of vehicles well. Moreover, the trust management mechanism of IoT based on the blockchain usually adopts the architecture of a single chain, which will trigger many transactions and encounter node hardware bottlenecks. Therefore, how to design a more efficient trust management mechanism and a more comprehensive evaluation system of vehicle nodes is still a challenging problem.

### 2.4. Combination of Double-Layer Blockchain and Trust Management

Lee et al. [19] proposed a two-layer blockchain trust management model for the Internet of Vehicles, which is composed of the local one-day message blockchain and the global vehicle reputation blockchain. The data in the global vehicle reputation blockchain are generated by RSUs located in different regions, which consist of the vehicle’s reputation score based on the vehicle’s historical behavior. Therefore, each vehicle’s reputation is updated and permanently stored in the global vehicle reputation blockchain for further query. In the local one-day message blockchain, vehicles and RSUs store and share local traffic information in a short period of time. RSUs and vehicles in the same region act as blockchain nodes. This blockchain creates a new block at a set time every day and deletes the previously recorded blockchain data. The system model in [19] calculated the credibility of the message by voting. However, the voting system assigns every voter the same weight and fails to restrict the behavior of voters, which cannot resist voting attacks such as manipulation, control, and bribery.

Kandah et al. [20] also proposed a two-layer blockchain trust management model composed of platoon blockchain and global blockchain. The participating nodes of a platoon blockchain are a group of vehicles with a small gap in proximity and speed. They store the localized trust consensus (trust value of vehicles), while the global blockchain stores the trust factors of all vehicles in the system, that is, the data in the platoon blockchain is added to the global blockchain through mining. In the mining stage, RSU mines the block using the trust bidding system. However, Kandah et al. [20] does not give a specific method to evaluate the vehicle, and any vehicle can access the trust value of another vehicle from anywhere, which will cause privacy leakage of the vehicle and other problems. The difference between single-layer blockchain and double-layer blockchain is shown in Table 1. The shortcomings of various trust management mechanisms are shown in Table 2.

## 3. Trust Management Mechanism of Double-Layer Blockchain: System Model, Design and Implementation

Consider a scenario where vehicle A needs to know about the traffic and business situation on street A. Vehicle A sends a request to the nearby RSU (we assume that each vehicle is equipped with an Onboard Unit (OBU), which uses Dedicated Short Range Communication (DSRC) or Cellular-V2X (C-V2X) communication technology for micro-wave communication with the RSU). Upon receiving the request, the RSU queries the trust value of vehicle A and allows vehicle A to use the service if its trust value is above a certain threshold. RSU queries relevant data in the RSU blockchain and returns it to vehicle A through a secure transmission channel. Vehicle A can then fully understand the situation of the front area according to the data returned by RSU and the data in its own vehicle blockchain.

However, vehicles and RSUs can both become malicious nodes and act maliciously, affecting other nodes in the system. For example, malicious RSUs may tamper with vehicle trust values, and malicious vehicles may send false messages. To solve the problem of malicious nodes, we propose a trust management mechanism based on the double-layer blockchain. This mechanism is divided into three parts. Of which, the first part is the double-layer blockchain, the second part is the system architecture, and the last part is the consensus mechanism. We introduce the proposed double-layer blockchain trust management mechanism through these three parts.

### 3.1. System Model

**Figure 1:**
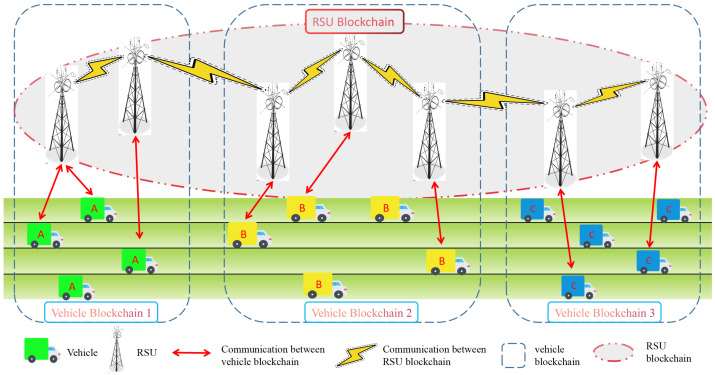
DLBTM system model.

In this section, we introduce the system model and participants of double-layer blockchain trust management, as shown in Figure 1. The list of notations is shown in Table 3.

#### 3.1.1. Double Layer Blockchain

Our DLBTM model, as shown in Figure 1, consists of the vehicle blockchain and the RSU blockchain. Specifically, the vehicle blockchain is a consortium blockchain. The participating nodes of the vehicle blockchain are a large number of vehicles and a small number of RSUs. When the vehicle detects an event, such as a blockage in the road ahead, it generates a message and sends a message to the RSU to report the incident. After receiving the message, the RSU determines whether the message is important. Important messages refer to messages that are related to driving efficiency and safety, such as the message reported by the vehicle to the RSU informing of traffic congestion in the front area. If the message reported by the vehicle to the RSU is redundant data from its own sensors, we believe that such messages are not as important.

Unimportant messages, such as data from the vehicle sensors, vehicle speed, and vehicle direction, are stored in the vehicle blockchain. This design has two advantages.

1. It can reduce the memory cost for RSUs. If the RSU blockchain stores these unimportant messages, it will cause a waste of storage resources;
2. It can provide fast message sharing and speed up consensus process building. By storing these messages with no value to other regions, but value to the local region on the vehicle blockchain can help other vehicles in the same region receive the message faster, and help them reach a consensus more quickly.

For important messages, such as road congestion ahead, RSUs temporarily store these important messages locally. Then, according to Algorithm 1, RSUs use TextRank [21] algorithm to generate message digest for important messages. In summary, the main purpose of the vehicle blockchain is to store unimportant messages generated by the vehicle (deleted every 1 h).

The RSU blockchain is a private blockchain in which RSUs from different geographical locations participate as blockchain nodes. For example, all RSUs in a city join the RSU blockchain as blockchain nodes. The vehicle blockchain is a consortium blockchain, with pre-selected nodes being RSUs, which participate in consensus, and where the data are stored by RSUs. The RSU blockchain is a private blockchain, and data are also stored by RSUs. When vehicles and RSUs join the system, they need to register with a trusted institution, which will manage the vehicle blockchain and RSU blockchain. The data stored in the blockchain will naturally be managed by the trusted institution. Although RSUs have a better storage capacity compared to vehicles, there are also limitations to RSU storage, which can be improved by upgrading hardware facilities to RSUs.

In summary, the RSU blockchain stores the trust value of all vehicles and the summary of important messages generated by the evaluated vehicles, which is convenient for future vehicles to request corresponding information according to their needs and interests to obtain services.

#### 3.1.2. Participants in the System Model

The entire double-layer blockchain system model comprises RSU and vehicle nodes.

- RSUs: The RSUs are responsible for block generation, vehicle final trust calculation, offering messages to vehicles that send a request, storing summaries of important messages, and storing unimportant messages for a period of time;
- Vehicles: Responsible for message generation, message evaluation, and message requests.

### 3.2. System Design and Implementation

**Figure 2:**
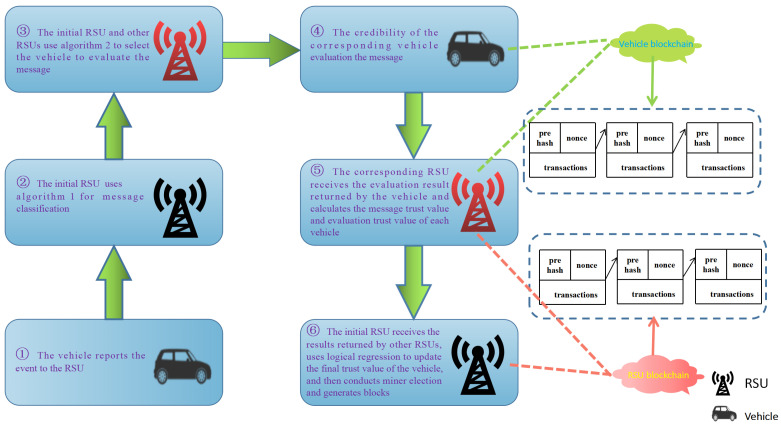
DLBTM system process.

The DLBTM system architecture is shown in Figure 2. Each specific time, for example, 30 s, each RSU calculates locally whether it can become a miner. We denote the RSU that becomes a miner of the initial RSU. First, the initial RSU runs Algorithm 1 to process important messages it has stored but has not yet written into the blockchain. After processing, the initial RSU sends the classified summary of important messages to other RSUs. The initial RSUs and other RSUs then run Algorithm 2 to select the vehicle that is most similar to the message being evaluated for message evaluation. The initial RSUs and other RSUs then send the classified summary of an important message to the selected vehicle of their own vehicle blockchain. Vehicles use Equation (1) to calculate message credibility and then return to the corresponding RSU. After receiving the calculation result, the corresponding RSU uses the average value to obtain the message credibility for evaluating different vehicles for the same message, and records the difference between the credibility of a message evaluated by a vehicle and its average value. The signature is returned to the initial RSU. After receiving all the results, the initial RSU regroups the messages, that is, sends the messages with the same vehicle ID into a group to facilitate the calculation of the trust value of the vehicles. The vehicle node has two identities, one as the sender of the message and the other as the evaluator of the message. As a message sender, when other vehicle nodes evaluate the message sender’s message, they will obtain the message sender’s message trust value. As a message evaluator, when evaluating the message sent by other vehicle nodes, they will obtain the message evaluator’s evaluation trust value, and the vehicle node has the historical trust value. Combining these three values, the initial RSU uses logistic regression to generate the vehicle’s final trust value. The initial RSU generates the corresponding block and sends it to other RSUs. If other RSUs recognize this block, mining will be carried out after this block.

#### 3.2.1. System Initialization

The vehicle node and RSU node of the DLBTM system model proposed in this paper use elliptic curve cryptography (ECC) for encryption and decryption, and the vehicle and RSU signatures use the ECC signature algorithm.

- RSU Registration: $RSU_{i}\;\left(i=1,\;\ldots ,\;N\right)$ selects an elliptic curve $y^{2}=x^{3}+ax+b$, and randomly selects a point as the base point P. $RSU_{i}$ selects a large number $d_{i}\in Z_{q}^{*}$ as the private key and generates the public key $Q_{RSU_{i}}=d_{i}P$. $RSU_{i}$ sends public key $Q_{RSU_{i}}$ to Certificate Authority (**CA**) for registration, and **CA** issues a certificate to $RSU_{i}$.
- Vehicle Registration: $vehicle_{j}\;\left(j=1,\;\ldots ,\;M\right)$ selects an elliptic curve $y^{2}=x^{3}+mx+n$, and randomly selects a point as the base point P. $vehicle_{j}$ selects a large number $d_{j}\in Z_{q}^{*}$ as the private key and generates the public key $Q_{vehicle_{j}}=d_{j}P$. $vehicle_{j}$ sends public key $Q_{vehicle_{j}}$ and private key $d_{j}$ to **CA** for registration, and **CA** issues certificate to $vehicle_{j}$.

Encryption and decryption of vehicles and RSUs: Vehicles and RSUs that need to send message **M** first randomly select a number *k* to generate ciphertext C=(kP,M+kQ) and send it to the corresponding receiver. After receiving ciphertext *C*, the receiver uses private key *d* to decrypt it, i.e., M=M+kQ−dkP.

#### 3.2.2. Message Classification

RSU classifies messages periodically using Algorithm 1. First, it loops through messages stored by itself but not yet written into blocks. For each message, RSU checks the message type for sorting. The messages are added to the same list if they are of the same type. Then add them to the TypeList at the end of step 1. For each element in the TypeList, the element’s messages are of the same type. In this case, the TextRank algorithm is used to generate the message summary, and the generated message summary is added to the EvalList and sent to other RSUs.
**Algorithm 1** Message Classification on Type**Input:**  $msg=\{msg_{i}^{1},\;msg_{i}^{2},\;\ldots ,msg_{i}^{n},\;msg_{j}^{1},\;msg_{j}^{2},\;\ldots ,\;msg_{j}^{n},\;\ldots ,\;msg_{k}^{m}\}$,           TypeList={$TypeList_{1}$, $TypeList_{2}$, …, $TypeList_{n}$}.**Output:** $EvalList=\{SumList_{1},\;SumList_{2},\;\ldots ,\;SumList_{n}\}$.1:**Step 1:** Message classification2:**for** each $message_{k}^{m}\in msg$ **do**3:   Check the type of message (there is this field in the message);4:   Then classify according to the type of $message_{k}^{m}$;5:   Add $message_{k}^{m}$ to $TypeList_{n}$;6:   **if** msg == NULL **then**7:      Stop classifying message;8:   **end if**9:**end for**10://TypeList is a two-dimensional list;11:**Step 2:** Message summary list generation12:**for** each $TypeList_{j}$∈TypeList **do**13:    generate a summary of $TypeList_{j}$. i.e., TextRank Algorithm.14:    add the summary to $SumList_{i}$;15:    Generate $EvalList=\{SumList_{1},\;SumList_{2},\;\ldots ,\;SumList_{n}\}$;16:**end for**17:send EvalList to all other RSUs.

#### 3.2.3. Selecting Message Evaluator

After other RSUs receive the EvalList (a classified list of important message summaries), the initial RSU and other RSUs use the message evaluator selection algorithm as shown in Algorithm 2 to select the appropriate evaluator vehicle for message evaluation.
**Algorithm 2** Message Evaluator Selection**Input:**  $EvalList=\{SumList_{1},\;SumList_{2},\;\ldots ,\;SumList_{n}\}$.            $VehList=\{Vehicle_{1},Vehicle_{2},\;\ldots ,Vehicle_{m}\}$.**Output:**  $EvaluatorList=\{EvaluatorList_{1},\;EvaluatorList_{2},\;\ldots ,\;EvaluatorList_{n}\}$.1:**for** each $Vehicle_{i}\in VehList$ **do**2:   Data processing by using Simhash algorithm;3:   Calculate Hamming distance of $Vehicle_{i}$ and EvalList;4:   **if** Hamming distance ($Vehicle_{i}$, EvalList) < ⊝ **then**5:       Add $Vehicle_{i}$ to EvaluatorList;6:   **end if**7:**end for**8:Return EvaluatorList.

First, we loop through each vehicle in the VehList. Then, we use the Simhash algorithm [22] to process EvalList and the vehicle, obtaining the Hamming distance of the vehicle and EvalList. If the Hamming distance between the two was less than the threshold, it was considered that the two are similar and meet the evaluation conditions. Therefore, the vehicle is then added to EvaluatorList.

#### 3.2.4. Message Trust Value of Vehicle

After the initial RSU and other RSUs send the evaluation request to the vehicle, the vehicle receives the message to be evaluated, and uses the following Equation (1) to calculate the credibility of the message.

(1) $$C_{i}^{j,k}=\alpha_{1}e^{-bd_{j,k}}\;+\;\alpha_{2}t_{j,k}\;+\;\alpha_{3}\frac{Num_{type}}{Num_{all}}+\;\alpha_{4}Sim_{i}^{j}$$

$C_{i}^{j,k}$ means that $vehicle_{i}$ evaluates the credibility of the $message_{k}$ sent by $vehicle_{j}$, of which $\alpha_{1},\;\alpha_{2},\;\alpha_{3},\;\alpha_{4}$ is the coefficient, and $\alpha_{1}+\alpha_{2}+\alpha_{3}+\alpha_{4}=1$, where *b* is the weight coefficient used to control the weight of $d_{j,k}$ in the calculation. $d_{j,k}$ represents the distance between the sender’s $vehicle_{j}$ and the place where $event_{k}$ occurred. This means the farther the distance is, the lower the credibility of the message. $t_{j,k}$ represents the time used to report this message minus the time of the first occurrence of this message, which is the freshness of the message. $\frac{Num_{type}}{Num_{all}}$ represents the proportion of the type of this message in the total evaluated message. The higher the proportion, the higher the credibility, $Sim_{i}^{j}$ refers to the similarity between $vehicle_{i}$ and $vehicle_{j}$. If $vehicle_{i}$ has recently released similar messages or $vehicle_{i}$ and $vehicle_{j}$ have similar driving tracks, then the similarity is high.

When the vehicle has evaluated all the messages, the result is returned to the corresponding RSU. After the corresponding RSU receives the result, the average value of messages sent by each evaluated vehicle is calculated, as shown in Equation (2)

(2) $$AvgScore_{j}^{t}=\frac{\sum_{i=1}^{n}\sum_{k=1}^{m}\omega_{i}^{t}C_{i}^{j,k}}{m*n}$$

where $AvgScore_{j}^{t}$ represents the message trust value of $vehicle_{j}$ as the message sender in time *t*, $\omega_{i}^{t}$ represents the trust value of $vehicle_{i}$ as the weight of the score, and the numerator is the weighted sum of the scores of different vehicles on each message of $vehicle_{j}$, and the denominator represents the total number of times that other vehicles evaluate the messages of $vehicle_{j}$ (i.e., m ∗ n). The result of dividing the two is $AvgScore_{j}^{t}$, which represents the average score of messages sent by $vehicle_{j}$ in time *t*.

#### 3.2.5. Evaluation Trust Value of Vehicles

When the vehicle acts as a message evaluator, its behavior also needs to be constrained and evaluated, and, therefore, its trust value as a message evaluator needs to be evaluated.

Therefore, when the corresponding RSU receives the evaluation result returned by the vehicle, we need to compare it with results returned by different vehicles. After receiving all the results for the same message, the corresponding RSU obtains the average score of the same message and then records the difference between the msg of the same message evaluated by each vehicle and the average. Finally, the sum of the value difference of each vehicle in this evaluation stage is obtained, denoted as ϕ. At the same time, record the number of times the vehicle is evaluated in this evaluation phase count and the time spent in this evaluation phase time. Thus, the trust value of the vehicle as the message evaluator in the evaluation stage is obtained, as shown in Equation (3).

(3) $$e_{i}^{t}=\eta_{1}\frac{count}{time}+\eta_{2}e^{-\beta \phi }$$

of which, $\eta_{1}$ and $\eta_{2}$ are weight coefficients, and $\eta_{1}+\eta_{2}=1$, $\frac{count}{time}$ is the number of messages evaluated by vehicles in time *t*, that is, the activity, ϕ is the sum of the differences between the vehicle’s evaluation time *t* and the average. The larger the sum, the smaller the $e^{-\beta \phi }$ value, and the smaller the vehicle’s evaluation trust value. After the corresponding RSU completes these tasks, it returns the results to the initial RSU.

#### 3.2.6. Vehicle Final Trust Value Update

After the initial RSU receives the results returned by the other RSUs, logistic regression is used to update the trust values of the vehicles. Specifically, we define the final trust value of a vehicle as the probability that a vehicle provides satisfactory service to other vehicles or RSUs [23]. The service here includes the sending and evaluating of a message. As the sender of a message, satisfactory service refers to sharing correct, objective, and timely information. While as the evaluator of a message, it refers to returning objective evaluation results to RSUs.

Specifically, in the subsequent stage, when $vehicle_{i}$ requests interested messages from RSU, $vehicle_{i}$ may or may not be satisfied with the messages provided by $vehicle_{j}$. If $vehicle_{i}$ is satisfied, then $vehicle_{j}$ is credible with high probability. Otherwise, $vehicle_{j}$ is incredible with high probability. We use $q_{i,j}^{t}$ to denote the quality of service provided by $vehicle_{j}$ to $vehicle_{i}$ in time *t*. Since the message of $vehicle_{j}$ may not only provide services to $vehicle_{i}$, we use $q_{j}^{t}=q_{i,j}^{t}⋃q_{i,j}^{t}$(m≠i) to indicate the quality of service provided by $vehicle_{j}$ to other vehicles and $vehicle_{i}$. Three factors affect the trust value of the vehicle, namely the trust value when the vehicle is the message sender, the trust value when the vehicle is the message evaluator, and the historical final trust value of the vehicle (at time slot t−1). We use the vector $\gamma^{t}={\left[\gamma_{avgscore}^{t}+\gamma_{e}^{t}+\gamma_{history}^{t}\right]}^{T}$ to represent the above three factors, then the final trust value is the probability of $vehicle_{j}$ providing satisfactory service to other vehicles at time *t*. Namely

(4) $$E\left[q^{t}|\gamma^{t},\chi \right]=\rho^{t}=\frac{1}{1+e^{-\gamma^{t}\chi }}$$

specifically, we use the vector $T={\left[1,2,3,\;\ldots ,t\right]}^{T}$ to represent the past time period. For the parameter χ, we use the initial given value, $\gamma =\gamma^{t}\left(t=1,2,\;\ldots ,t\right)$, and use Equation (4) to compute the expectation $\rho^{t}$, that is, the E step of the EM algorithm. For the expectation $\rho^{t}$, we use the maximum likelihood estimation parameter χ to obtain the new parameter $\chi_{new}$, which is the M step of the EM algorithm. Finally, we calculate $E\left[q^{t+1}|\gamma^{t+1},\chi \right]=\rho^{t+1}=\frac{1}{1+e^{-\gamma^{t+1}\chi_{new}}}$ and reach the final trust value of $vehicle_{j}$ at time t+1, namely $\rho^{t+1}$.

### 3.3. Consensus Mechanism

In general, we need to increase the probability of RSUs with more data and can sense the changes in vehicle reputation value more sensitively to obtain the right to publish blocks. Specifically, if a vehicle’s trust value changes a lot, so does the veracity of the information the vehicle reports. Moreover, if the size of the data stored in an RSU increases dramatically over a period of time, it will mean a lot of new events are happening in that area. Therefore, it is important to ensure that vehicles with large trust changes and RSUs with large data storage are updated first, which is critical to the security and timeliness of the IoV.

#### 3.3.1. Consensus Mechanism of Vehicle Blockchain

In the vehicle blockchain of our DLBTM system, we use Ouroboros [24] as the consensus protocol of the vehicle blockchain, which is based on POW. We use the number of non-important messages stored in RSUs as a stake to design the mining difficulty. The greater the stake held by RSUs, the greater the probability of it becoming the leader.

We delete unimportant messages every 1 h, and divide 1 h into several periods, and each period into multiple rounds. During each round, we generate no more than 1 block, and every hour the RSUs in the vehicle blockchain will create the genesis block of the new blockchain. In the vehicle blockchain, the participant in the consensus is the RSU in the vehicle blockchain, but the vehicle does not participate in the consensus process.

Next, we will take a closer look at the selection process of the leader at a given time in the vehicle blockchain.

In $round_{j}$, each RSU independently calculates whether it is the leader or not. Specifically, each RSU uses the $F(S,\varepsilon ,{slot}_{j})$ function and its own stake ratio to calculate the leader of $round_{j}$. The probability to be selected as the leader is expressed by the following Equation (5):

(5) $$P_{m}=\frac{RSU_{m}}{\sum_{k=1}^{n}RSU_{k}}$$

of which $RSU_{m}$ is the stake of RSUs, $P_{m}$ represents the proportion of $RSU_{m}$’s stake in all RSUs of vehicle blockchain.

F in $F(S,\;\varepsilon ,{slot}_{j})$ is a function that can be implemented using the follow-the-satoshi algorithm, $S=\left(PK_{1},\;s_{1}\right),\;\left(PK_{2},\;s_{2}\right),\;\ldots ,\;\left(PK_{m},\;s_{m}\right)$ is the stake distribution of all RSUs in the vehicle blockchain, where $PK_{1}$ and $s_{1}$ are the public key and its stake of $RSU_{1}$ respectively, ε is the random number seed in the current period, $slot_{j}$ is the current $round_{j}$.

The remaining rounds, as the above, are an iterative process, and each round elects its leader independently. In addition to selecting the leader, multiple “endorsement nodes”, the verifiers of the transaction, need to be selected. The endorsement node verifies whether the transaction is legitimate and sends the legitimate transaction to the leader.

#### 3.3.2. Consensus Mechanism of RSU Blockchain

We adopt the Ouroboros Praos [25] protocol as the consensus protocol of the RSU blockchain in our proposed DLBTM system. The reasons we use Ouroboros Praos in the RSU blockchain instead of Ouroboros, like in the vehicle blockchain are two-fold.

1. The nodes of the vehicle blockchain participating in consensus are pre-selected RSUs, and the transactions of the vehicle blockchain are relatively less important data. Even if attackers know the leaders in advance, bribery attacks and DDOS attacks on them will have little impact on the stability of the DLBTM system;
2. The RSU blockchain stores important information and trust value shared by vehicles, so the identity information of the leader needs to be hidden. The nodes involved in mining in the RSU blockchain run verifiable random functions (VRFS) locally and only know who is the leader when other nodes receive the block, thus, reducing the impact of an attack on the DLBTM system.

We then elaborate on the leader selection process during a certain period in the RSU blockchain.

In $round_{i}$ of a certain period, each RSU participating in mining runs a verifiable random function (VRF) locally. The method for RSU to judge whether it is a leader is to determine whether the generated random number is lower than a threshold, the value of which is related to the ratio of node stake (i.e., Equation (6)). Therefore, there may be multiple RSUs as leaders in $round_{i}$. However, it is also possible to have no leader at all, and the probability of becoming a leader is as follows:

(6) $$P_{n}=\phi_{f}\left(RSU_{i}\right)=1-{\left(1-f\right)}^{RSU_{i}}$$

where $RSU_{i}$ refers to the stake proportion of $RSU_{i}$, and f is a parameter of the Ouroboros Praos protocol. In particular, $RSU_{i}=1$, i.e., $\phi_{f}\left(1\right)=f$, represents the probability that $RSU_{i}$ node holds all the stake and is selected as the leader. The function is not a linear function.

### 3.4. Security Analysis

1. **Resist malicious RSUs:** Based on the Ouroboros protocol cluster, our DLBTM model can effectively defend against malicious RSUs. RSUs are important nodes in both the vehicle blockchain and the RSU blockchain. If normal RSUs are hacked and become malicious RSUs, they cannot construct a false block. This is because the consensus protocol used by the vehicle blockchain and RSU blockchain is the Ouroboros protocol cluster, and malicious RSUs as the leader can only create a blank block, since the transactions in the block are verified by multiple endorsement nodes and then sent to the leader. At the same time, according to the Ouroboros protocol cluster, the probability of becoming the leader is proportional to the proportion of the node’s stake, and even the RSU with a relatively high proportion of the intrusion stake cannot forge the block. If an attacker controls multiple RSUs to become malicious nodes, the Ouroboros protocol cannot issue fake blocks as long as the malicious RSUs do not hold more than 51% of the blockchain;
2. **Defense against false message attacks:** Our DLBTM model can effectively defend against false message attacks. In false message attacks, an attacker may invade vehicle nodes, turning normal vehicle nodes into malicious ones. The malicious vehicle node then sends a false message to the RSU in an attempt to influence other nodes in the system. However, in DLBTM, other vehicle nodes will calculate the trust of false information, and, therefore, grant the malicious node a very low message trust value, so that when RSU updates the final trust value of the malicious node, the malicious node will receive a very low final trust value. Therefore, when other vehicle nodes request service later, RSU will choose the message provided by the vehicle node with a higher trust value. Therefore, false information published by malicious nodes will not be selected by RSUs, so the malicious nodes cannot affect other nodes in the system. In the end, malicious nodes that continue to publish false information will be excluded from the system;
3. **Defence against false score attacks:** Our DLBTM model system can effectively defend against false score attacks. In IoV, malicious nodes may be selected by RSUs as message evaluators according to their similarity, generating false scores for messages of normal vehicle nodes, thus, affecting the whole system. However, in our DLBTM, RSU selects multiple message evaluators when scoring the message. According to Equation (3), RSU will record the difference between the message score evaluated by the malicious node and the mean value, and identify the possible malicious nodes, reducing their final trust value. In this way, malicious nodes will be excluded from our system, and fail to undermine the system. Furthermore, the number of malicious nodes is often limited. Therefore, those malicious nodes are usually unable to evaluate the same message because RSUs select vehicles as message evaluators based on similarity. Therefore, our DLBTM can reduce the possibility of collusion attacks;
4. **Data integrity:** Our DLBTM model system can effectively prevent important data such as trust values and important message summaries of vehicles from being tampered with by malicious nodes to ensure data integrity. In DLBTM, both unimportant messages recorded in the vehicle blockchain and important message summaries and trust values recorded in the RSU blockchain are created by the leader and then completed by endorsement nodes that add legitimate transactions to the block, which become a consensus of all RSUs. The sequence and content of the blocks are protected using hash chains. The hash value of each block is unique. Once any content of any block is modified, the hash of the other blocks will be changed [26]. Therefore, a malicious RSU node that intends to tamper with the data and modify a vehicle’s trust value will have to modify the data in the current block and recalculate the previous block’s hash value on the chain, which is impossible in practice. Therefore, our DLBTM can effectively protect data integrity.

## 4. Experiment and Analysis

To verify the validity of the proposed system model, we use IoV network simulation platforms **SUMO1.11.0**, **OMNET++5.7**, and **Veins 5.2**. Specific parameters are shown in Table 4.

### 4.1. Time Complexity and Space Complexity Analysis

In this section, we compare the complexity of our DLBTM with DTMS [27], a popular blockchain-based trust management model. DTMS considers message classification and message evaluator for trust value calculation, and the message classification algorithm of DTMS only generates indexes for classified messages, while does not generate a message summary. This results in wasted time and storage space during message evaluation by vehicle nodes in subsequent phases. Therefore, DTMS has higher time complexity in terms of message classification and message evaluator selection compared with our DLBTM.

The comparison of time complexity and space complexity between DTMS and DLBTM message classification algorithms is shown in Table 5. We count the number of statements executed in the algorithm to do so. There is a ‘while’ loop and a ‘for’ loop in DTMS’s message classification algorithm, and the number of statements executed is quadratic to the data size, and, therefore, the time complexity is $O(n^{2})$. However, the message classification algorithm of DLBTM has only one ‘for’ loop, therefore, the time complexity is O(n). Therefore, Algorithm 1 in DLBTM executed faster due to the computation of the lower order. However, the temporary storage space occupied by the two algorithms during operation is the same, and, therefore, the space complexity of both algorithms is $O(n^{2})$.

The comparison of the time complexity and space complexity of the DTMS and DLBTM message evaluator selection algorithms is shown in Table 6. There are two ‘for’ loops in DTMS’s message evaluator algorithm selection. The number of statements executed in the loop is quadratic with the increase in data size, so the time complexity is $O(n^{2})$. However, DLBTM’s message evaluator algorithm proposed in this paper, namely, Algorithm 2, has an O(n) time complexity. This means that the computational complexity required to execute the algorithm in DLBTM is lower than in DTMS. However, the temporary storage space used by the two algorithms during operation is the same, and, therefore, the space complexity of DTMS and DLBTM is both O(n).

### 4.2. Simulation Results and Performance Evaluation

**Figure 3:**
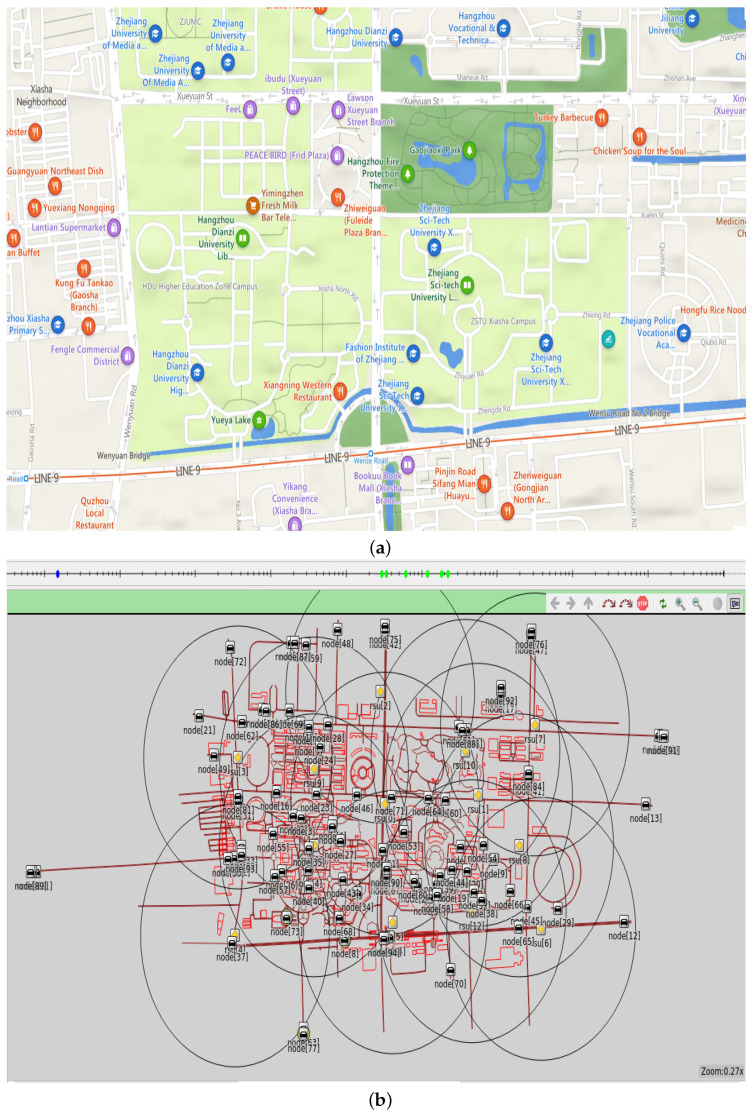
(**a**) Road map near Hangzhou Dianzi University. (**b**) Scene simulation map was drawn with OMNET++ and SUMO.

We design a virtual traffic environment using Veins and simulate traffic scenes within 2 km of Hangzhou Dianzi University. Figure 3a shows the situation of roads and streets near Hangzhou Dianzi University, and Figure 3b shows the simulation map drawn by sumo and OMNET++ based on Figure 3a. The black vehicle icons on the simulated map in Figure 3b represent vehicle nodes, the yellow diamond icons represent RSUs, and the large black circles represent the communication range of RSUs. There are 100 or 50 vehicles on this simulated map, which report messages to nearby RSUs at regular intervals, and then proceed with the process in Figure 2. We set the initial value of the final trust value of the vehicle to 0.5, which means that the newly registered vehicle has a 50% probability of providing satisfactory service to other vehicles, which is easy to formulate using the sigmoid function in logistic regression. We set the system to conduct trust management by RSU every 30 s, and the entire simulation time 160 s, with vehicle nodes reporting messages to RSU at any moment.

**Figure 4:**
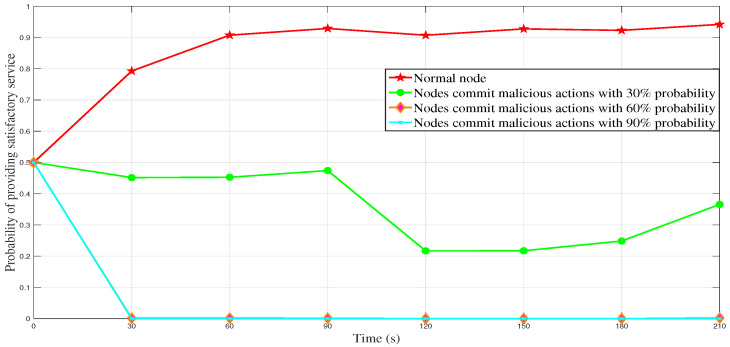
The final trust value of malicious behavior made by nodes with different probabilities (he total number of vehicles is 50).

**Figure 5:**
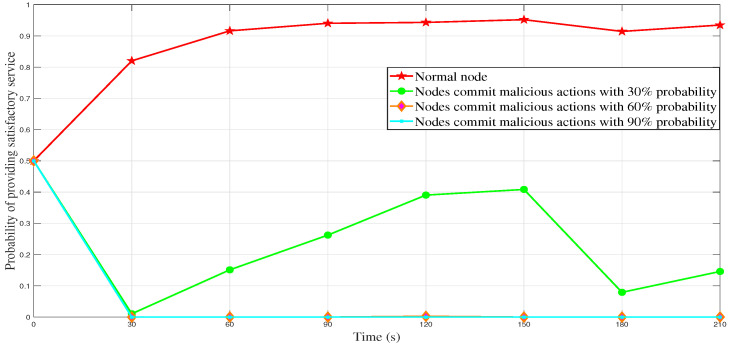
The final trust value of malicious behavior made by nodes with different probabilities (the total number of vehicles is 100).

Figure 4 and Figure 5 show the changes in the final trust value caused by malicious behavior by a vehicle node with different probabilities over time when the total number of vehicles during the simulation period is 50 or 100. It can be seen that as a normal node, the final trust value of the vehicle (that is, the probability of providing a satisfactory service) approaches 1. This means that the normal node actively reports the perceived events to the RSU, and the system assigns them a higher trust value based on their positive history behavior since their reported events are accurate and effective. Therefore, the system is more probable to believe the information reported by this vehicle node will be very satisfactory to other vehicle nodes in the next period. Specifically, suppose the vehicle node has a 30% probability of malicious behavior. In that case, the final trust value of it fluctuates between 0 and 0.5, which is quite different from the final trust value of the normal node and is easy to discriminate. If the vehicle node has a 60% probability of malicious behavior, and the final trust value of the vehicle is close to 0, this indicates that the probability of providing a satisfactory service is close to 0. If there is a 90% probability that the vehicle node will commit malicious behaviors, the final trust value of the vehicle node approaches 0 infinitely, indicating that it is difficult to provide satisfactory services to other nodes in the future. It can be seen that the reliability of our DLBTM has been fully verified. Regardless of whether malicious nodes act maliciously with high probability or low probability, or whether traffic is congested or unblocked, our proposed DLBTM model can identify malicious nodes well.

**Figure 6:**
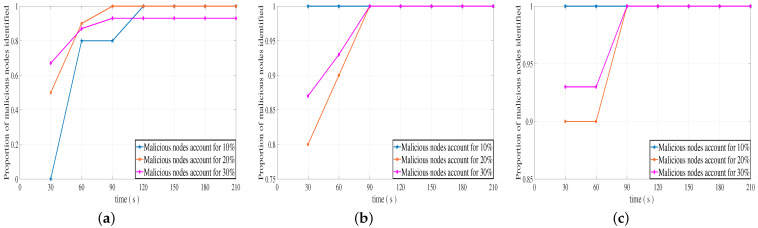
Identification rate of malicious nodes with different proportions (vehicles: 50). (**a**) A 30% probability of malicious behavior, (**b**) a 60% probability of malicious behavior, and (**c**) a 90% probability of malicious behavior.

Figure 6a–c shows the changes in the proportion of malicious nodes identified by our DLBTM when the total number of vehicles is 50, that malicious nodes of the vehicle commit malicious behavior with different probabilities, and that there are malicious nodes with different proportions in the system. It can be clearly seen from Figure 6a, that our DLBTM recognizes more than 80% of malicious nodes in the second stage around 60 s when malicious nodes perform malicious behaviors with a probability of 30%, and the recognition rate is above 90% as time goes by. As shown in Figure 6b, when malicious nodes commit malicious behaviors with a probability of 60%, at about 30 s in the first stage, the proportion of malicious nodes identified by the proposed system model is above 80%, and the recognition rate reaches 100% over time. As shown in Figure 6c, when malicious nodes commit malicious behaviors with a 90% probability, the proportion of malicious nodes identified by the proposed system model is above 90% at about 30 s of the first stage, and the recognition rate reaches 100% with time.

**Figure 7:**
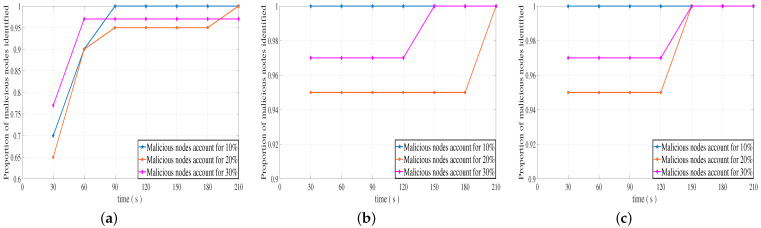
Identification rate of malicious nodes with different proportions (vehicles: 100). (**a**) A 30% probability of malicious behavior, (**b**) a 60% probability of malicious behavior, and (**c**) a 90% probability of malicious behavior.

Figure 7a–c shows the changes in the proportion of malicious nodes identified by our DLBTM when the total number of vehicles is 100, malicious nodes of the vehicle commit malicious behavior with different probabilities, and there are malicious nodes with different proportions in the system. It can be clearly seen from Figure 7a, that our DLBTM recognizes more than 90% of malicious nodes in the second stage around 60 s when malicious nodes perform malicious behaviors with a probability of 30%, and the recognition rate approaches 100% as time goes by. As shown in Figure 7b, when malicious nodes commit malicious behaviors with a probability of 60%, at about 30 s in the first stage, the proportion of malicious nodes identified by the proposed system model is above 95%, and the recognition rate reaches 100% over time. As shown in Figure 7c, when malicious nodes commit malicious behaviors with a 90% probability, the proportion of malicious nodes identified by the proposed system model is above 95% at about 30 s of the first stage, and the recognition rate reaches 100% with time.

The comparison between Figure 6 and Figure 7 shows the effect of changes in the probability of malicious behavior and the number of vehicles. When the probability of malicious behavior among nodes in the system is 30% and the total number of vehicles is 50, our DLBTM system identifies malicious nodes at a rate of over 92% over time. When the total number of vehicles is 100, our DLBTM system identifies malicious nodes at a rate of over 97% over time. When the probability of malicious behavior among nodes in the system is 60% and the total number of vehicles is 50, our DLBTM system’s recognition rate of malicious nodes reaches 100% over time. When the total number of vehicles is 100, our DLBTM system’s recognition rate of malicious nodes reaches 100% over time. When the probability of malicious behavior among nodes in the system is 90% and the total number of vehicles is 50, our DLBTM system’s recognition rate of malicious nodes reaches 100% over time. When the total number of vehicles is 100, our DLBTM system’s recognition rate of malicious nodes reaches 100% over time. In short, when the probability of malicious behavior is constant, the increase or decrease in the number of vehicles has little impact on the proportion of DLBTM systems identifying malicious nodes.

In summary, regardless of whether the proportion of malicious nodes in the system is 10%, 20%, 30%, or the total number of vehicles is 50 or 100, our DLBTM can accurately identify the proportion of malicious nodes with a probability more than 90%.

**Figure 8:**
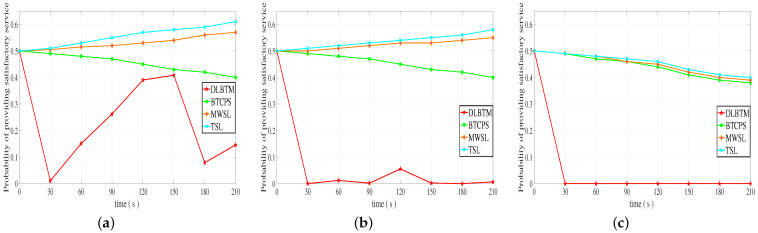
The change in the final trust value of a malicious vehicle over time. (**a**) The probability of malicious behavior is 30%, (**b**) the probability of malicious behavior is 50%, and (**c**) the probability of malicious behavior is 80%.

We compared our DLBTM with the BTCPS model [28], MWSL method [13], and TSL method [29]. We consider the impact of the probability Pr of malicious behavior by a malicious vehicle. When Pr = 30%, Pr = 50%, and Pr = 80%, we observe the change in the final trust value of a malicious vehicle. As shown in Figure 8, when Pr = 30%, both DLBTM and BTCPS can reduce the trust value of the vehicle, but BTCPS does not reduce the trust value as quickly as DLBTM, which means it cannot quickly identify malicious nodes. When Pr = 50%, both DLBTM and BTCPS can reduce the trust value of the vehicle, but DLBTM recognizes malicious nodes significantly faster than BTCPS. When Pr = 80%, all four methods can reduce the trust value of the vehicle, but DLBTM is significantly faster than the other three methods in identifying malicious nodes.

In the trust management mechanism of the IoV based on a single blockchain, vehicle nodes in the system generate a large number of transactions, and vehicle nodes process and store a large amount of transaction information. Therefore, vehicle nodes may encounter hardware bottlenecks. However, in a double-layer blockchain, due to the stronger storage and computing power of RSUs compared to vehicles, we store data on RSUs, reducing the burden on vehicles. At the same time, our algorithm uses message summarization to reduce the storage space of RSU data, effectively improving the effectiveness of data storage and sharing between vehicles and RSUs, thereby indirectly improving the throughput of the system.

## 5. Conclusions

In this paper, we propose a double-layer blockchain-based trust management mechanism DLBTM to solve the malicious attacks targeted on the communication among vehicles and RSUs in IoV. Using the double-layer blockchain, we can reduce the burden of vehicles in IoV, realize hierarchical management of nodes in IoV, protect vehicular privacy, implement hierarchical data storage and sharing, and realize effective trust evaluation and management of vehicle nodes. Remarkably, our message classification on type algorithm and message evaluator selection algorithm has lower time complexity compared with similar algorithms, and simulation experiments show that our trust management mechanism can effectively identify malicious nodes. Therefore, our DLBTM is effective and feasible in complex IoV environments. For future research, we will introduce an incentive mechanism into our model to promote cooperative behavior.

## Figures and Tables

**Table 1:** Difference between the single and double-layer blockchain.

Summary	Description
The single blockchain and double-layer blockchain have different participating nodes	The participating nodes of a single blockchain are vehicles and RSUs, and the miners may be vehicles or RSUs, while the participating nodes of a vehicle blockchain in a double-layer blockchain are vehicles and RSUs; the RSU blockchain only has RSU participating nodes, and the miners are RSUs.
The difference in functionality between the single blockchain and double-layer blockchain	We separate the three tasks of data storage, message evaluation, and trust value calculation. The vehicles in the vehicle blockchain perform message evaluation, while the RSU of the RSU blockchain performs data storage and trust value calculation. The nodes in the single blockchain need to complete the above three tasks
The length of time the single blockchain and double blockchain store data is different	The nodes in the single blockchain need to store all historical data. The vehicle blockchain in the double-layer blockchain is a temporary blockchain (deleted every hour, that is, creating a new Genesis block and deleting previously recorded blockchain data). The RSU blockchain is a permanent blockchain that stores important message summaries and vehicle trust values.

**Table 2:** Comparison of the shortcomings of various trust management mechanisms.

Trust Management Mechanism	Shortcoming
Centralized Trust Management	All data are stored and processed on a central server, which is generally located in the cloud and far from the vehicle, and may not meet the latency requirements of the IoV. At the same time, there are also the single points of failure, concurrency problems (at the same time, a large number of vehicles access the central server), big data (storage and transmission of massive data), and other problems.
Distributed Trust Management	In distributed trust management mechanisms, the task of managing and storing data is usually completed by vehicles or RSUs. However, vehicles and RSUs are usually distributed outdoors, and security measures are not as strict as central servers. RSUs have the possibility of failure, and security and reliability are not fully guaranteed.
Combination of Blockchain and Trust Management	Vehicles can query the trust data of another vehicle anytime and anywhere, which can cause privacy breaches of the vehicle. The current research does not have a reasonable plan for the collection, storage, and management of trust data. Vehicles need to store a complete blockchain ledger, which will increase the storage burden on vehicle nodes. Due to the varying importance of data generated by vehicles, the system does not differentiate the importance of messages and instead stores and shares data.
Combination of Double-layer Blockchain and Trust Management	The system model in reference [19] calculates the credibility of messages by voting on other vehicles but does not constrain the behavior of voters, making it easy to launch attacks on them. Reference [20] does not provide a specific method for evaluating vehicle trust values.

**Table 3:** Main notations used in this paper.

Notation	Description
$RSU_{i}$	The i-th RSU in IoV
$vehicle_{j}$	The j-th vehicle in IoV
P	Point generator of an additive cyclic group $G_{q}$
$d_{i}$	A private key of $RSU_{i}$
$Q_{RSU_{i}}$	A public key of $RSU_{i}$
$d_{j}$	A private key of $vehicle_{j}$
$Q_{vehicle_{j}}$	A public key of $vehicle_{j}$
**M**	Messages sent by RSU or vehicles
msg	List of each message sent by each vehicle
TypeList	A two-dimensional list of messages grouped by type
EvalList	Message summary list EvalList generated from TypeList
$message_{k}^{m}$	The m-th message reported by $vehicle_{k}$ to the nearby RSU
VehList	A list containing many vehicles
EvaluatorList	List of vehicles selected for evaluated messages

**Table 4:** Experimental environment.

Parameters	Settings
Simulation map area	2500 m × 1500 m
Number of RSUs	13
Number of vehicles	100 or 50
Vehicle speed	60 km/h
Vehicle communication distance	300 m
RSU communication distance	600 m
Vehicle transmit power	10 mW
RSU transmit power	30 mW
Mac protocol	IEEE802.11p

**Table 5:** Algorithm 1 analysis.

Model	Time Complexity	Space Complexity
DTMS	$O(n^{2})$	$O(n^{2})$
DLBTM	O(n)	$O(n^{2})$

**Table 6:** Algorithm 2 analysis.

Model	Time Complexity	Space Complexity
DTMS	$O(n^{2})$	O(n)
DLBTM	O(n)	O(n)

## Data Availability

Due to privacy issues, we are unable to provide data.
